# Body Condition Peaks at Intermediate Parasite Loads in the Common Bully *Gobiomorphus cotidianus*

**DOI:** 10.1371/journal.pone.0168992

**Published:** 2016-12-28

**Authors:** Alberto Maceda-Veiga, Andy J. Green, Robert Poulin, Clément Lagrue

**Affiliations:** 1 Department of Integrative Ecology, Estación Biológica de Doñana-CSIC), Sevilla, Spain; 2 Institute of Research in Biodiversity (IRBio), Faculty of Biology, University of Barcelona, Barcelona, Spain; 3 Department of Wetland Ecology, Estación Biológica de Doñana-CSIC, Sevilla, Spain; 4 Department of Zoology, University of Otago, Dunedin, New Zealand; University of Pretoria, SOUTH AFRICA

## Abstract

Most ecologists and conservationists perceive parasitic infections as deleterious for the hosts. Their effects, however, depend on many factors including host body condition, parasite load and the life cycle of the parasite. More research into how multiple parasite taxa affect host body condition is required and will help us to better understand host-parasite coevolution. We used body condition indices, based on mass-length relationships, to test the effects that abundances and biomasses of six parasite taxa (five trematodes, *Apatemon* sp., *Tylodelphys* sp., *Stegodexamene anguillae*, *Telogaster opisthorchis*, *Coitocaecum parvum*, and the nematode *Eustrongylides* sp.) with different modes of transmission have on the body condition of their intermediate or final fish host, the common bully *Gobiomorphus cotidianus* in New Zealand. We used two alternative body condition methods, the Scaled Mass Index (SMI) and Fulton’s condition factor. General linear and hierarchical partitioning models consistently showed that fish body condition varied strongly across three lakes and seasons, and that most parasites did not have an effect on the two body condition indices. However, fish body condition showed a highly significant humpbacked relationship with the total abundance of all six parasite taxa, mostly driven by *Apatemon* sp. and *S*. *anguillae*, indicating that the effects of these parasites can range from positive to negative as abundance increases. Such a response was also evident in models including total parasite biomass. Our methodological comparison supports the SMI as the most robust mass-length method to examine the effects of parasitic infections on fish body condition, and suggests that linear, negative relationships between host condition and parasite load should not be assumed.

## Introduction

By definition, parasites are organisms that benefit from their hosts as a habitat and food source, causing some degree of harm to them that, in extreme cases, can lead to host death and host populations to collapse [1]. Typically, acute host mortality occurs in infections caused by parasites with direct life-cycles, including ectoparasitic protists [2,3] and the flatworms *Gyrodactylus* spp. [4]. These parasites are some of the most ubiquitous aquatic infectious agents and include the invasive *Gyrodactylus salaris* that has devastated Atlantic salmon (*Salmo salar*) stocks in Norway [5]. However, rapid lethal effects are rare in hosts infected by parasites with complex life-cycles, such as cestodes and trematodes [1]. Nonetheless, chronic infections can cause major health issues to the hosts, including castration [6], energy depletion [7] and morphological malformations [8]. As a given host-parasite combination often shows different pathological outcomes under the same conditions [9], a better knowledge of the relationship between parasitic infections and host condition will improve our understanding of host-parasite co-evolution.

Exposure to challenging conditions, including parasites, triggers a cascading metabolic response in individuals, altering many physiological variables such as differential white blood cell count, body water and glycogen content, tissue isotopic composition or stress and reproductive hormones that can be used as biomarkers of an animal’s body condition (e.g. [10,11,12]). Measuring biomarkers, however, requires practitioners to have additional training and can also be costly, time consuming or require prohibitively rapid sample processing in the field [13]. These limitations may explain the popularity among ecologists of body condition indices (BCIs) based on mass-length relationships [14,15,16]. These determine condition based on an animal’s weight while adjusting for difference in structural size (e.g. length), with the scaled mass index (SMI) [16,17] and the analysis of covariance (ANCOVA [18]) being two popular and powerful non-lethal methods. SMI values are, however, easier to interpret as they represent the weight of each individual at a standardised body size [17]. Thus, the SMI offers a powerful, non-destructive tool to assess how infections can influence host body condition.

The SMI has been demonstrated to be suitable for addressing body condition in fish, amphibians, birds, mammals and insects [19,20,21,22]. However, no BCI should be assumed to accurately reflect ‘true condition’ without analysing body composition and the response of the BCI to specific indicators of disease [23,24]. In the context of BCIs based on mass-length relationships, an animal’s body condition is generally defined as the energy capital stored in the body as a result of feeding [17]. This assumes that animals in poor condition ingest less food or food of poorer quality than healthy conspecifics [17]. However, any factor altering an animal weight or shape can influence BCIs [23]. The biomass of the parasites themselves can also contribute to raising the BCI of the host [25].

Parasites are usually assumed to be deleterious for their hosts [26,27], and have typically been linked to hosts with poor body condition (e.g. [27,28]). Body condition is a key parameter for fisheries management [29,30], as it can act as a proxy for predicting future fish growth and reproductive success [31]. Alterations in BCIs are more likely to occur in endoparasitic infections, as endoparasites can cause energy depletion and abnormal growth of host internal tissues (e.g. [32,33]). Also, these parasites often induce complex behavioural alterations and shifts in habitat use in intermediate hosts that may serve to increase parasite transmission probabilities to final hosts, concomitantly increasing intermediate host predation risk and prey intake in definitive host predators [33]. Since these changes can boost host weight including energy reserves, endoparasitic infections are a suitable case-study to test for potential neutral or positive effects of parasites on BCIs.

In this study, we explore in detail the consequences of endoparasitic infections for fish BCIs. We examined relationships between BCIs and abundances of six parasite species (five trematodes and one nematode) with different modes of transmission in the common bully *Gobiomorphus cotidianus* McDowall 1975, in three New Zealand lakes over three seasons. We consider how the effects of parasites depend on their abundance, biomass, and life cycle, and test the assumption prevailing in the literature that there is a negative, linear relationship between body condition and parasite load. We also consider how condition varies among seasons and among lakes. The common bully is an abundant native, benthic fish, widely distributed in New Zealand, which feeds mainly on crustaceans in Lake Waihola and on chironomid larvae in Lakes Hayes and Tomahawk. This fish is an important food resource for bird and fish species, such as introduced trout (*Salmo trutta* Linnaeus, 1758 and *Oncorhynchus mykiss* Walbaum, 1792) and native eels (*Anguilla* spp.). The common bully hosts a variety of both juvenile and adult helminth parasites [25]. Its maximum size is 150 mm total length but it tends to be smaller in lakes (70–80 mm, [34]). The life cycle of lake populations of common bullies is largely unknown, but multiple spawning is likely to occur from September to March in all three lakes (CL *pers*. *observ*.). We use the SMI as an index of fish body condition. For comparison, we also used Fulton’s condition factor (CF) because it is still widely used in fish condition studies (e.g. [35,36,37,38]), even though CF violates several key assumptions [16,18]. In particular, CF is a simplistic ratio that assumes a cubic relationship between mass and length that does not reflect fish growth patterns in nature.

## Materials and Methods

### Ethical Note

This study was approved by the University of Otago Animal Ethics Committee, which limited the sample size and provided the guidelines for euthanasia and fish sampling (Ref: OT-34204-RES and ET 10/2012).

### Study area and fish sampling

The common bully was used to test for potential effects of parasites on fish body condition. Fish were sampled (n = 235, but see below) in three lakes on New Zealand’s South Island, Hayes (44°58'S, 168°48'E), Waihola (46°01‴S, 170°05'E) and Tomahawk (45°54'S, 170°33'E), in three austral seasons: early spring (September), summer (January), and late autumn (May). All lakes are eutrophic but especially Lake Hayes due to urban sewage discharges and runoff of nutrients from adjacent agricultural areas. Another major difference among the three lakes is the high turbidity of Lake Waihola due to its exposure to strong winds. Combinations of fish catching gear types were used so that accurate cross sections of common bully size classes were sampled from each lake in each season. First, 8 fyke nets and 40 minnow traps were set in the evening along the littoral zone of the lakes. The next day, trapped fish were recovered and killed for later dissection. Fish sampling was then complemented using a standard, fine-mesh purse seine net (5 mm mesh size). Fish were killed immediately by severing the spinal cord with scissors and destroying the brain stem. Death was instantaneous and confirmed by the absence of eye reflex and operculum movements. Fish were then stored on ice to preserve internal tissues and parasites for identification.

In the laboratory, fish were measured to the nearest mm (total length), weighed to the nearest 0.01 g (total fish mass) and then dissected. The digestive tract and all internal organs and tissues were removed and preserved in 70% ethanol for later diet and parasite analyses. Eviscerated fish bodies were frozen individually until dissection. Complete necropsies of all fish were conducted under a dissecting microscope as described in Lagrue & Poulin [25]. The head, gills, eyes, brain and spine of each fish were examined using fine forceps to pull apart fish tissues and obtain a precise estimation of parasite load for each fish. Soft tissues (muscle and skin) were removed from the spine, crushed between two glass plates and examined by transparency to identify and count parasites. Internal organs and tissues and the gut were first rinsed in water to wash off the ethanol. The gut was then separated from other organs and tissues. Liver, swimbladder, gall bladder, gonads and other organs and tissues from the body cavity (fat, mesentery, kidneys and heart) were all screened for parasites. Finally, the gut was dissected, its contents removed, screened for parasites and then set aside for diet examination. Oesophagus, stomach, intestine and rectum were then examined for gut parasites. All parasites were identified, counted and a subsample of 20 individuals per species (or all individuals when < 20 were found in a fish) were measured to the nearest 0.01 mm (diameter for spherical parasites, such as encysted trematode metacercariae; length, width and thickness for flattened ellipsoids, like adult trematodes; length and width for cylindrical parasites such as nematodes).

### Scaled mass index calculation

All fish below 50 mm were excluded from the analyses because their body mass was considered unreliable (n = 122). These individuals are more likely to be subjected to bias owing to the relatively high contribution of water droplets and the gut content from the last meal to the mass of these small fish, and they deviated markedly from the line fitted between body weight and length. We also subtracted parasite biomass from fish weight because these parasites can represent a high proportion of fish biomass [25].

The scaled mass index (SMI) was used as an index of body condition (BCI) following Maceda-Veiga et al. [22], and was calculated as: SMI = Wi [L0/Li]^bSMA, where W_i_ and L_i_ are the weight and length of each specimen respectively, L_0_ is a suitable length to which the BCI values are standardized, and b_SMA_ is the scaling exponent, i.e. the slope of a standardised major axis (SMA) regression of the mass-length relationship. In our case, for L_0_ we used the arithmetic mean of the data-set analysed for the fish host (75.4 mm). To compute the b_SMA_, we applied an SMA regression to log_10_-transformed weight and length values to determine the slope of the fitted line (i.e. b_SMA_). At this step, the criterion to remove outliers was maximising the better refit (R^2^) of the regression line. Individuals that were outliers were then returned to the sample when SMI was calculated (see [17] for details). SMI results were also compared with those of the Fulton’s condition factor (CF = W_i_ / L_i_^^3^ x 10^5^). We were unable to use the ANCOVA method as an alternative BCI because of heterogeneity of slopes (fish length-season interaction P < 0.05, see [39]).

### Relationships between body condition, parasite load and fish size

The relative effect of parasite abundance (number of parasites in infected and uninfected hosts) on the scaled mass index (SMI) and Fulton’s condition factor (CF) as body condition measures in each individual fish was examined using a generalised linear model (GLM). Initial GLMs included linear and quadratic parasite abundances, but only significant quadratic terms are shown in the final models. Since there are major differences in mean mass among parasite taxa (*Apatemon* sp., 0.103 mg; *Stegodexamene anguillidae* Macfarlane, 1951, 0.478 mg; *Telogaster opisthorchis* Macfarlane, 1945, 0.091 mg; *Tylodelphys* sp., 0.032 mg; *Eustrongylides* sp., 4.169 mg; and *Coitocaecum parvum* Crowcroft, 1945, 0.052 mg), additional GLMs were built using linear and quadratic parasite biomass. To test for overall effects of parasites on fish body condition, independent GLMs were also built using total parasite abundance or biomass as predictors. We used a Gaussian error distribution and log_10_-transformation for continuous variables to reduce data dispersion and improve linearity. Lake identity was included as a fixed factor in the models because variables affecting host abundance, such as primary productivity, vary among lakes [40]. Although these authors [40] also found that the abundance of these six trematode taxa did not vary markedly across seasons in New Zealand, season was included as a fixed factor in the models to account for possible seasonal effects on host condition. We also compared parasite abundance and fish size (length) across lakes and seasons using GLMs with log-transformed data and quasipoisson and Gaussian error distribution, respectively, followed by Tukey’s Honest Significant Difference (HSD) post hoc tests. As *Tylodelphys* sp. only occurred in Lake Hayes, its mean abundance was only compared among seasons. Models were validated with q-q plots of residuals and by plotting fitted vs. predicted values. Explained variation (R^2^) in GLMs was calculated as follows: (null deviance–residual deviance)/null deviance.

To complement the results of GLMs, we performed a series of hierarchical partitioning analyses (HP) on the variables retained as having a significant effect in the GLMs, using the same error distribution. Although causality cannot be determined in observational studies, an advantage of HP is that it can disentangle the effect of a unique factor (e.g. lake effects) from that of the rest of predictors (e.g. parasite effects) [41]. In contrast to GLM, HP assumes equal slopes of relationships for different lakes and seasons. Other modelling criteria such as AIC and model averaging are discouraged because collinearity results in biased parameter estimates [42]. We assessed the significance of HP models using a randomization test for hierarchical partitioning analysis. Significance in HP analysis was based on the upper 0.95 confidence interval, but it was reached at *P* < 0.05 in the remaining statistical procedures. Non-linear relationships between parasite load and BCIs were explored visually using lowess regressions (function ‘lowess’ with default settings).

Pair-wise correlations among abundances of different parasite taxa, and between parasite abundance and fish size (length) were examined using Spearman’s rank correlation coefficient (r). *Stegodexamene anguillidae* was removed for the GLM models to avoid collinearity, since its abundance was highly correlated with that of two other species (r≥0.7). All statistical analyses were performed in R version 3.1.1 [43], using stats, MASS, car [44], lmodel2 [45], rand.hp [46] and multicomp libraries.

## Results

### Body condition measures

The common bully (n = 113) showed a nonlinear relationship between weight (mean±SE: 7.04±0.66; min-max: 1.30–23.02 g) and length (75.4±7.1; 50–113 mm) that was linearized by log-transformation (R^2^ = 0.99). In contrast to Fulton’s condition factor’s (CF) assumption of isometry (i.e. that the scaling exponent between log weight and log length is 3), the b_SMA_ value was 2.88 (confidence intervals 2.86–2.91) indicating that the weight-length relationship was negatively allometric.

### Relationships between fish size, parasite load and body condition indices across lakes and seasons

Mean fish length varied across lakes (F_2,104_ = 15.92; P<0.001), with the lowest values observed in Lake Waihola (Tukey’s HSD, all P<0.05). Six species of metazoan parasites (five trematodes and one nematode) were recovered in sufficient numbers to be included in the analyses (Table 1). The common bully is the final host for one species only, the trematode *Coitocaecum parvum*. The four species of trematodes that use the common bully as a second intermediate host all contact the fish as free-swimming infective stages (cercariae), penetrate through the skin or gills, migrate to various internal tissues and encyst as metacercariae, except *Tylodelphys* sp. which does not encyst and moves freely within fish eyes. *Coitocaecum parvum* and the nematode *Eustrongylides* sp. are both acquired when fish consume an infected prey (amphipod and oligochaete, respectively); the trematode matures as an adult worm and lives freely within the fish gut, whereas the nematode encysts in the gut wall or mesentery and never migrates to the musculature or viscera.

**Table 1:** Parasite species considered in this study, their known life cycles and mean size (±S.D.) in common bullies.

Parasite taxa	Parasite size				Hosts		
	Length (mm)	Width (mm)	Thickness (mm)	Diameter (mm)	1st intermediate	2nd intermediate	Definitive
*Apatemon* sp. (T)	-	-	-	0.58±0.02	Snail, *Potamopyrgus antipodarum*	Fish, including *Gobiomorphus cotidianus*	Various water birds (herons, gulls, cormorants)
*Stegodexamene anguillae* (T)	-	-	-	0.81±0.28	Snail, *Potamopyrgus antipodarum*	Fish, including *Gobiomorphus cotidianus*	Eels, *Anguilla* spp.
*Telogaster opisthorchis* (T)	-	-	-	0.55±0.04	Snail, *Potamopyrgus antipodarum*	Fish, including *Gobiomorphus cotidianus*	Eels, *Anguilla* spp.
*Tylodelphys* sp. (T)	1.20±0.05	0.25±0.04	0.18±0.04	-	Unknown snail	Fish, including *Gobiomorphus cotidianus*	Crested grebe, *Podiceps cristatus*
*Coitocaecum parvum* (T)	1.04±0.21	0.36±0.07	0.22±0.04	-	Snail, *Potamopyrgus antipodarum*	Amphipods	Fish, including *Gobiomorphus cotidianus*
*Eustrongylides* sp. (N)	36.08±0.89	0.30±0.09	-	-	Oligochaetes	Fish, including *Gobiomorphus cotidianus*	Cormorants, *Phalacrocorax* spp.

T = trematode; N = nematode.

**Fig 1:**
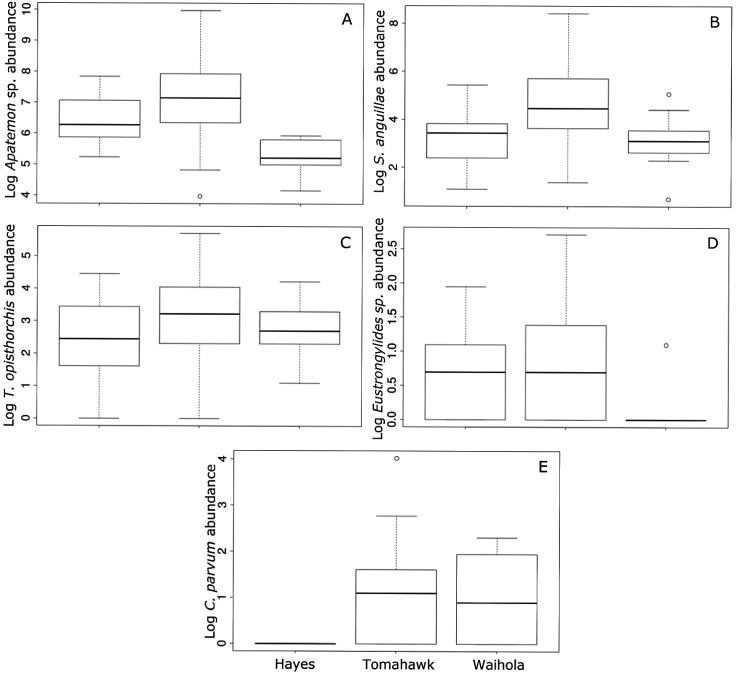
Abundance of *Apatemon* sp. (A), *Stegodexamene anguillae* (B), *Telogaster opisthorchis* (C), *Eustrongylides* sp. (D) and *Coitocaecum parvum* (E) in common bullies (*Gobiomorphus cotidianus*) collected in the different lakes. Each box corresponds to 25th and 75th percentiles; the dark line inside each box represents the median; error bars show the minima and maxima except for outliers shown as open circles.

Mean parasite abundance differed among lakes for all parasite taxa, with the exception of *T*. *opisthorchis* (Table 2). Fish in Lake Waihola had the lowest mean abundance of *Apatemon* sp. and *Eustrongylides* sp., whereas Lake Tomahawk had the highest abundance of *S*. *anguillae* (Fig 1). *Tylodelphys* sp. only occurred in Lake Hayes. Abundances of all six parasite taxa were mostly positively correlated (Table 3), especially that between *S*. *anguillae* and *Apatemon* sp. (r = 0.71) and between *S*. *anguillae* and *T*. *opisthorchis* (r = 0.70). No seasonal effects were observed for mean fish length (F_2,104_ = 0.01; P = 0.59) or the abundance of any of the six parasite taxa (all P>0.05).

**Table 2:** Results of GLM models for comparisons of mean abundance for the six parasite taxa found in common bullies (*Gobiomorphus cotidianus*) across three lakes and seasons in New Zealand. The trematode *Tylodelphys* sp. only occurred in Lake Hayes and was excluded from cross-lake comparisons. Bold values indicate significance at P< 0.05.

	Likelihood ratio test	Degrees of freedom	P-value
*Apatemon* sp.			
Lake	20.87	2	**<0.01**
Season	0.05	2	0.97
Lake x Season	6.55	4	0.16
*Stegodexamene anguillae*			
Lake	11.64	2	**<0.01**
Season	1.84	2	0.40
Lake x Season	6.83	4	0.14
*Telogaster opisthorchis*			
Lake	3.12	2	0.21
Season	1.08	2	0.58
Lake x Season	0.97	4	0.91
*Tylodelphys* sp.			
Season	2.04	2	0.36
*Eustrongylides* sp.			
Lake	9.30	2	**<0.01**
Season	0.21	2	0.89
Lake x Season	2.78	4	0.59
*Coitocaecum parvum*			
Lake	19.34	2	**<0.01**
Season	0.00	2	1.00
Lake x Season	3.59	4	0.47

**Table 3:** Spearman rank correlation coefficients (r) between abundances of the six parasite taxa found in common bullies (*Gobiomorphus cotidianus*) across three lakes and seasons. For *Tylodelphys* sp., pair-wise correlations were only made for Lake Hayes, where this trematode was present. Bold values indicated significance at P< 0.05.

	1		2		3		4		5
	r	*P*	r	*P*	r	*P*	r	*P*	r	*P*
1. *Apatemon* sp.										
2. *Stegodexamene anguillae*	0.71	**<0.01**								
3. *Telogaster opisthorchis*	0.64	**<0.01**	0.70	**<0.01**						
4. *Tylodelphys* sp.	0.57	**<0.01**	-0.13	0.54	0.27	0.22				
5. *Eustrongylides* sp.	0.62	**<0.01**	0.58	**<0.01**	0.54	**<0.01**	0.40	0.06		
6. *Coitocaecum parvum*	0.08	0.42	0.23	**0.01**	0.19	0.05	nd	Nd	0.14	0.13

nd—*Tylodelphys* sp. and *C*. *parvum* did not co-occur in any of the three lakes

**Fig 2:**
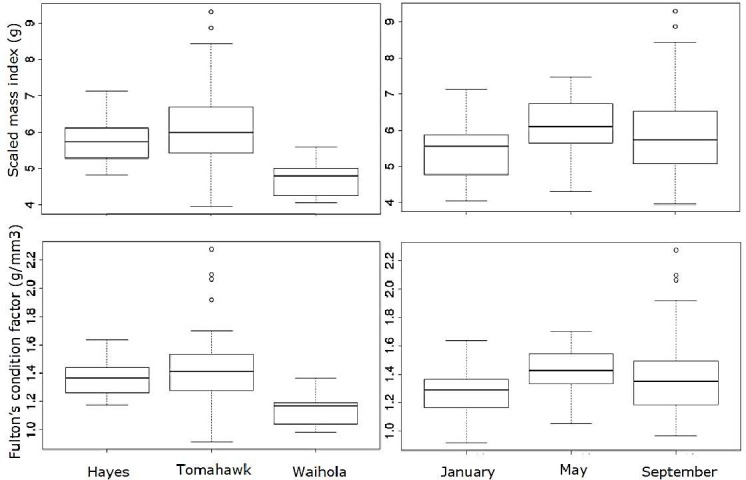
Scaled Mass Index (SMI, g) and Fulton’s condition factor (CF, g/mm^3^) for common bullies (*Gobiomorphus cotidianus*) collected in the three lakes over three seasons in New Zealand. Each box corresponds to 25^th^ and 75^th^ percentiles; the dark line inside each box represents the median; error bars show the minima and maxima except for outliers shown as open circles.

As parasite abundances were correlated, it was necessary to disentangle this relationship and analyse the independent and joint effects of the six parasite taxa on fish body condition indices (BCI). We analysed the effects of total parasite abundance and, in separate models, analysed effects of five of the taxa simultaneously, removing *S*. *anguillae* (see above). In all models, lake and season were the main determinants of fish body condition as defined by SMI and CF (Tables 4 and 5). In particular, the highest BCI values occurred in fish captured in May and in Lake Waihola across all three seasons (Fig 2). Sex ratio was 1:1 in all seasons and lakes, so was not a confounding factor. The results of GLM and HP models were mostly concordant, indicating that collinearity between predictors was of minor importance in our data-set (Tables 4 and 5). The only discrepancy observed between GLM and HP was when the latter retained the parasite *T*. *opisthorchis* as having a significant effect (Table 5). This species, however, made a low, independent contribution to the variance in BCI in the HP analysis compared to lake, season and *Apatemon* sp. abundance (Table 5).

**Table 4:** Results of GLM models for scaled mass index (SMI) and Fulton's condition factor (CF) in common bullies (*Gobiomorphus cotidianus*) that include lake, season and total or individual abundances of parasite taxa (from Table 3, excluding *S*. *anguillidae* owing to strong correlations with other taxa) as explanatory factors. Explained variation in all models is reported as a percentage (%). Only significant squared parasite abundances are shown. Bold values indicate significance at P< 0.05.

Body condition and predictors	SS	F	df	P-value	%
**SMI**					
Individual parasite effects					
Season	0.16	4.57	2,102	**0.013**	41.40
Lake	0.15	4.22	2,102	**0.017**	
*Apatemon* sp.	0.26	14.28	1,102	**0.0003**	
*Apatemon* sp. SQRD	0.28	15.62	1,102	**0.0001**	
*Telogaster opisthorchis*	0.07	4.04	1,102	**0.047**	
*Tylodelphys* sp.	0.01	0.44	1,102	0.51	
*Eutrongylides* sp.	0.05	2.94	1,102	0.09	
*Coitocaecum parvum*	0.01	0.41	1,102	0.52	
Total parasite effects					
Season	0.17	4.39	2,106	**0.015**	33.68
Lake	0.31	7.94	2,106	**0.001**	
All parasites	0.23	11.81	1,106	**0.001**	
All parasites SQRD	0.19	10.10	1,106	**0.002**	
**CF**					
Individual parasite effects					
Season	0.25	3.20	1,102	**0.044**	29.39
Lake	0.26	3.32	1,102	**0.040**	
*Apatemon* sp.	0.32	8.13	1,102	**0.005**	
*Apatemon* sp. SQRD	0.37	9.42	1,102	**0.003**	
*Telogaster opisthorchis*	0.05	1.23	1,102	0.269	
*Tylodelphys* sp.	0.02	0.45	1,102	0.503	
*Eutrongylides* sp.	0.05	1.33	1,102	0.251	
*Coitocaecum parvum*	0.005	0.13	1,102	0.719	
Total parasite effects					
Season	0.28	3.41	2,106	**0.037**	23.70
Lake	0.47	5.79	2,106	**0.004**	
All parasites	0.25	6.61	1,106	**0.015**	
All parasites SQRD	0.24	5.91	1,106	**0.017**	

SS = sum of squares, df = degrees of freedom

**Table 5:** Independent contribution (%) of lake, season and individual or total abundances of parasites retained as having a significant effect (from Table 4), to the explained variation of the hierarchical partitioning models performed on common bully (*Gobiomorphus cotidianus*) body condition as estimated by scaled mass index (SMI) and Fulton's condition factor (CF). Significance (in bold) was reached at the 95% confidence interval based on a randomized permutation test (see methods). Bold values indicate significance at P< 0.05.

Explanatory factors	SMI	CF
Individual parasite effects		
Season	**20.07**	**24.54**
Lake	**29.19**	**31.92**
*Apatemon* sp.	**23.63**	**20.87**
*Apatemon* sp. SQRD	**20.98**	**20.62**
*Telogaster opisthorchis*	**6.12**	2.04
Total parasite effects		
Season	**24.79**	**24.79**
Lake	**39.75**	**39.74**
All parasites	**19.85**	**19.85**
All parasites SQRD	**15.61**	**15.61**

**Fig 3:**
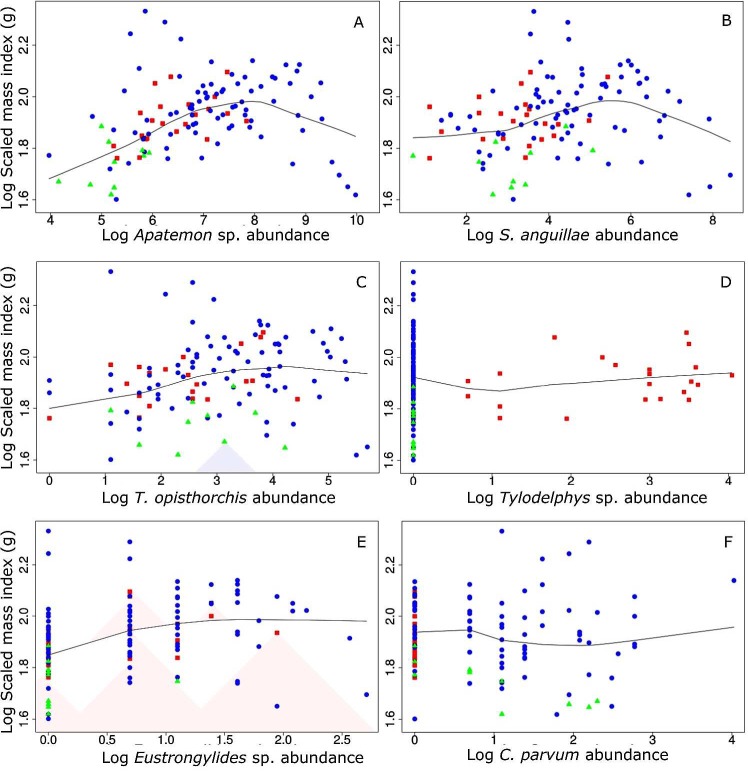
Relationships between scaled mass index (SMI, g) and abundances of the parasites *Apatemon* sp. (A), *Stegodexamene anguillae* (B), *Telogaster opisthorchis* (C), *Tylodelphys* sp. (D), *Eustrongylides* sp. (E) and *Coitocaecum parvum* (F) in common bullies (*Gobiomorphus cotidianus*) in lakes Hayes (red squares), Tomahawk (blue dots) and Waihola (green triangles). Lowess fitted curves are included on each graph.

**Fig 4:**
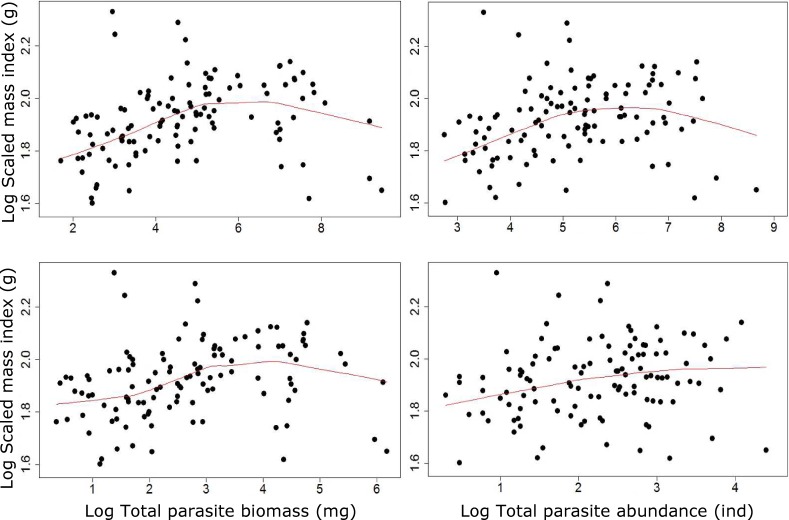
Relationships between scaled mass index (SMI, g) and total abundance (right) or biomass (left) of all six parasite taxa combined with (up) and without (down) the most abundant parasite taxa (*Apatemon* sp.) in common bullies (*Gobiomorphus cotidianus*). Lowess fitted curves are included on each graph.

There was a significant overall non-linear effect of the abundance of all six parasite taxa combined on *G*. *cotidianus* BCI, whether for CF or SMI (Table 4, Fig 3), although a higher proportion of variance in body condition was explained by the GLM for SMI (Table 4). Comparing the GLM model testing for the overall effects of all six parasite taxa combined on BCI with that including the abundance of individual parasite taxa suggests that the overall pattern was driven by the high abundance of *Apatemon* sp., which had a strong non-linear effect (Tables 4 and 5). The use of parasite biomass as a predictor instead of parasite abundance showed the same significant curvilinear relationships between fish body condition and parasite load (Fig 4, Table A in S1 File).

For both *Apatemon* sp. and *S*. *anguillae*, fish BCI increased to a threshold from which the relationship became negative (Fig 3). As the abundance of both these taxa was highly correlated, it was not possible to separate their individual effects on BCIs. When abundance of *Apatemon* sp. was removed from the total parasite load, there was no longer a significant curvilinear influence of parasite abundance on BCIs (Fig 4, Table B in S1 File). However, the significant curvilinear effect of total parasite biomass on fish body condition was retained after removing *Apatemon* sp. biomass (Fig 4, Table B in S1 File), which is likely to have been largely due to the influence of *S*. *anguillae* (Fig 3). All these effects of parasite loads on BCIs were detected because quadratic terms were included in the models to allow for non-linear effects. When parasite abundance was included in GLMs without quadratic terms, there were no detectable effects of parasites on BCI (Table C in S1 File).

**Fig 5:**
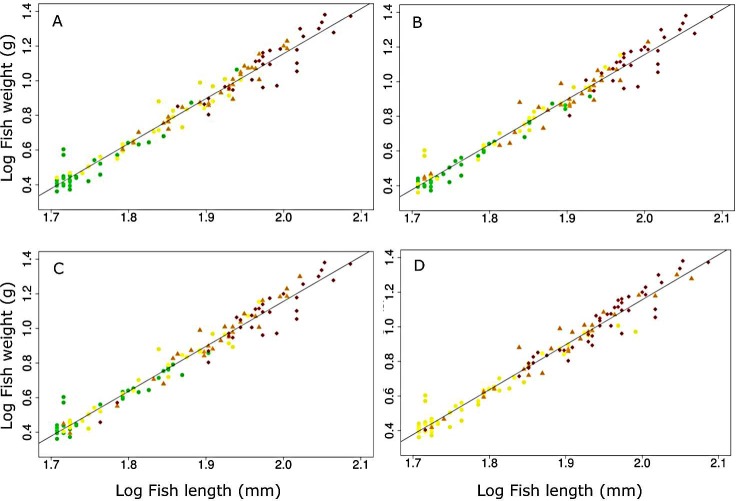
Relationships between fish size and weight in relation to abundances of the parasites *Apatemon* sp. (A), *Stegodexamene anguillae* (B), *Telogaster opisthorchis* (C), and *Eustrongylides* sp. (D). Abundance is grouped into quartiles (1^st^ Quartile: green triangles; 2^nd^ Quartile: yellow dots; 3^rd^ Quartile: brown squares, and 4^th^ Quartile: red diamonds). Zero abundance values comprised the first two quartiles of data for *Eustrongylides* sp. Plots are only shown for parasites whose abundances are highly correlated with fish size (r > 0.70) (see main text).

Parasite abundance significantly increased with fish body length in four parasite taxa (All r>0.70, P<0.01) (Fig 5), while there was no significant relationship for *C*. *parvum* (r = 0.14, P = 0.13) and *Tylodelphys* sp. (r = 0.25, P = 0.26).

## Discussion

Our study highlights the importance of considering non-linear responses when testing for the effects of parasites on host health, as defined by two body mass-length indices (BCIs) widely used to assess an animal’s body condition. BCIs varied strongly among lakes and seasons, but the effects of total parasite and *Apatemon* sp. abundance on the body condition of common bullies were only detected when quadratic terms were included in the models. A humpbacked relationship was also observed between BCIs and the biomass of all six parasite taxa combined, even when the most abundant taxa (*Apatemon* sp.) was excluded from the analysis. These results suggest that all six parasite taxa with different modes of transmission can have a cumulative effect on fish body condition. At the level of individual taxa, both trematodes *Apatemon* sp. and *S*. *anguillae* had similar curvilinear relationships with BCIs.

The relationship between the abundance of the six parasite taxa and the body condition of common bully was evident with the two methods used in our study (Fulton’s condition factor, CF; Scaled mass index, SMI). However, the SMI performed better than CF in our modelling approaches, as also recently reported for other fish species [22,47]. The CF does not adequately control for the changing relationship between length and mass during somatic growth. Nonetheless, our analyses using SMI and CF retained the same predictors as having a significant effect on the body condition of common bully, possibly because the scaling relationship assumed by the CF (i.e. W is proportional to L^3^) is reasonably close to the true scaling relationship (2.88), as previously suggested by Peig & Green [16]. This contrasts with Omar *et a*l. [47] who found pollution effects on the body condition of Nile tilapias (*Oreochromis mossambicus* Peters, 1852) using the SMI but not CF. In a preliminary analysis of a different data subset from the same host-parasite system, Lagrue & Poulin [25] found no relationship between total parasite load and fish body condition using the residual index (RI, [48]). The RI and other least square methods (OLS) including ANCOVA are less appropriate than those based on standardised major axis (SMA) such as the SMI, because the former do not deal with measurement error and natural variability in X and Y [23]. SMA and OLS values, however, are expected to be similar when the correlation coefficient between weight and length approximates to 1, as occurs in our study (r = 0.98). Thus, results of these alternative methods can be robust enough to be repeatable using the SMI, but when the correlation between weight and length is lower, SMA and OLS methods can give very different results [16,17]. So, the differences between the two studies are likely to be attributed to the different sets of fish individuals and parasite taxa used but, more importantly, to the fact that non-linear responses were not examined in Lagrue & Poulin [25].

Our results contrast with the viewpoint of most conservationists and livestock managers that helminth infections have pernicious effects on fish hosts [8,26]. However, their effects depend on many factors including parasite load [26,49], and our results for the trematode *Apatemon* sp. and total parasite abundance or biomass suggest that negative effects on body condition can be important at the highest levels. For instance, high numbers of large parasites such as the nematode *Eustrongylides* sp. and the eye fluke *Tylodelphys* sp. can severely alter tissue morphology and function, as they actively feed on the host [50,51]. However, the remaining parasite species are smaller and typically encyst in a dormant stage (metacercariae), causing relatively little damage to key host organs at low infection levels [48,52]. In fact, the positive relationship found between fish body condition and parasite abundance at low infection levels suggests that parasites can benefit from hosts with high energy reserves, and that parasites can promote an increase in body condition (see below). For example, infection of brine shrimps by cestodes dramatically increases the quantity of lipid stores in the host [53]. However, even at high parasite numbers, gross pathological effects were not observed during fish dissections in our study. Nonetheless, parasite effects may have been subtle or visible at other levels of biological organization (e.g. physiology, fecundity and behaviour [26,33], with ramifications for fish weight or shape and hence mass-length indices. It is also likely that parasites showing a neutral or even positive effect on fish body condition within the age class examined in our study would have had severe negative consequences if they had infected the host at an early life stage [8]. Likewise, the ability of infected hosts to face new stressors such as global change may have been altered by the parasites [9], as well as the performance of the host in the ecosystem [54].

In our study, differences among lakes were found to make the largest independent contribution to variation in body condition of common bully, with fish in Lake Waihola having poorer condition than those in Lakes Tomahawk and Hayes. This is likely to be mainly owing to differences in diet, as fish in Lakes Tomahawk and Hayes mostly fed on chironomids and those in Lake Waihola on amphipod crustaceans (Lagrue, *pers*. *observ*.), which are less nutritious [55]. A complementary explanation is that water turbidity in Lake Waihola reduced feeding rates of common bully, as demonstrated experimentally [56]. These two factors can also explain a smaller fish size in Lake Waihola which, in turn, had the lowest parasite numbers in our study, supporting evidence from previous studies that parasite load increases with fish size [57]. Another plausible explanation for our results is that local adaptation may have caused differences among lakes in fish size (e.g. [58]). The observed pair-wise correlations among abundances of most parasite taxa also suggest that these six parasite taxa are unlikely to strongly compete for host resources in mild infections, supporting previous observations for other similar parasites (e.g. [59]).

The relation between food intake and parasite load is likely to be highly complex. For example, a bold personality for a fish may increase food intake and at the same time the intake rate of infected intermediate hosts, making a negative correlation between BCIs and infection intensity less likely. As well as nutritional and growth implications *per se*, alterations in host feeding behaviour are likely to affect infection rates by trophically transmitted parasites [60]. On the other hand, parasites may manipulate their fish hosts and make them bolder so as to increase their encounter rate with final hosts such as piscivorous birds [33]. An increased foraging intake due to manipulation by parasites may counter and even exceed the negative effects of parasites on the size of energy stores, at least at low intensities as suggested by the humpbacked relationships for *Apatemon* sp. and *S*. *anguillae* (Fig 3). This is consistent with the manner in which the high infection intensity of *C*. *parvum* observed in fish captured in Lake Waihola (compared to values in the other two lakes) reflects their diet; fish mainly prey upon crustaceans in Lake Waihola and amphipod crustaceans are the intermediate host of *C*. *parvum* (Table 1). In addition, our results showed common bully to achieve better body condition in late autumn (May), possibly because fish increased energy reserves before wintering. Seasonal factors, however, did not influence intensity of any of the six parasite taxa, supporting previous findings by Lagrue & Poulin [44].

In conclusion, this study provides correlative evidence that parasite effects on the body condition of common bullies can range from positive to negative as parasite burden increases. Body condition measures based on mass-length relationships can, however, only detect major changes in weight or shape of an individual in relation to uninfected conspecifics [17,23]. Therefore, our findings need to be confirmed by future observational and experimental studies using other indicators of animal welfare, such as alterations in life-history traits [61] and blood biomarkers [13], including direct measures of energy reserves and immune cells activity [12]. Finally, our results suggest that previous studies relating host body condition and parasite load should be revisited to look for non-linear relationships to see if there is general support for our finding that body condition indices can be boosted at intermediate parasite loads.

## Supporting Information

S1 File Contains Tables A, B and C.
(DOCX)
