# Supplementary material for: Body Condition Peaks at Intermediate Parasite Loads in the Common Bully Gobiomorphus cotidianus
Source: PLoS One. 2016 Dec 28;11(12):e0168992. doi: 10.1371/journal.pone.0168992 (PMC5193454; doi:10.1371/journal.pone.0168992)
Supplement: S1 File — Contains Tables A, B and C. (DOCX) [file pone.0168992.s001.docx]

Table A. Results of GLM models for scaled mass index (SMI) and Fulton's condition factor (CF) in common bullies (*Gobiomorphus cotidianus*) that include lake, season and total or individual biomass of five parasite taxa (excluding *Stegodexamene anguillae* owing to strong correlations with other taxa, Table 3) as explanatory factors. Only significant squared parasite biomasses are shown. Bold values indicate significance at P< 0.05.

| Body condition and predictors | SS | F | df | P-value |
| --- | --- | --- | --- | --- |
| **SMI** |  |  |  |  |
| Individual parasite effects |  |  |  |  |
| Season | 0.157 | 4.35 | 2,102 | **0.015** |
| Lake | 0.125 | 3.45 | 2,102 | **0.035** |
| *Apatemon* sp. | 0.265 | 14.63 | 1,102 | **0.0002** |
| *Apatemon* sp. SQRD | 0.294 | 16.27 | 1,102 | **0.0001** |
| *Telogaster opisthorchis* | 0.048 | 2.70 | 1,102 | 0.103 |
| *Tylodelphys* sp. | 0.0055 | 0.30 | 1,102 | 0.582 |
| *Eutrongylides* sp. | 0.055 | 3.04 | 1,102 | 0.084 |
| *Coitocaecum parvum* | 0.00005 | 0.003 | 1,102 | 0.959 |
|  |  |  |  |  |
| Total parasite effects |  |  |  |  |
| Season | 0.171 | 4.61 | 2,106 | **0.012** |
| Lake | 0.225 | 6.06 | 2,106 | **0.003** |
| All parasites | 0.334 | 17.97 | 1,106 | **<0.0001** |
| All parasites SQRD | 0.275 | 14.77 | 1,106 | **0.0002** |
|  |  |  |  |  |
| **CF** |  |  |  |  |
| Individual parasite effects |  |  |  |  |
| Season | 0.241 | 3.06 | 2,102 | **0.057** |
| Lake | 0.231 | 2.94 | 2,102 | **0.050** |
| *Apatemon* sp. | 0.308 | 7.84 | 1,102 | **0.006** |
| *Apatemon* sp. SQRD | 0.372 | 9.47 | 1,102 | **0.003** |
| *Telogaster opisthorchis* | 0.029 | 0.74 | 1,102 | 0.391 |
| *Tylodelphys* sp. | 0.015 | 0.38 | 1,102 | 0.541 |
| *Eutrongylides* sp. | 0.051 | 1.30 | 1,102 | 0.256 |
| *Coitocaecum parvum* | 0.0003 | 0.008 | 1,102 | 0.927 |
|  |  |  |  |  |
| Total parasite effects |  |  |  |  |
| Season | 0.273 | 3.46 | 2,106 | **0.035** |
| Lake | 0.376 | 4.76 | 2,106 | **0.010** |
| All parasites | 0.376 | 9.53 | 1,106 | **0.002** |
| All parasites SQRD | 0.364 | 9.24 | 1,106 | **0.003** |

SS = sum of squares, df = degrees of freedom

Table B. Results of GLM models for scaled mass index (SMI) and Fulton's condition factor (CF) in common bullies (*G. cotidianus*) that include lake, season and total abundance or biomass of parasite taxa without *Apatemon* sp. as explanatory factors. Bold values indicate significance at P< 0.05.

| Body condition and predictors | SS | F | df | P-value |
| --- | --- | --- | --- | --- |
| **SMI** |  |  |  |  |
| Total parasite effects (abundance) |  |  |  |  |
| Season | 0.252 | 5.95 | 2,106 | **0.0035** |
| Lake | 0.422 | 9.96 | 2,106 | **0.0001** |
| All parasites | 0.050 | 2.41 | 1,106 | 0.1238 |
| All parasites SQRD | 0.017 | 0.82 | 1,106 | 0.3685 |
|  |  |  |  |  |
| Total parasite effects (biomass) |  |  |  |  |
| Season | 0.208 | 5.39 | 2,106 | **0.0059** |
| Lake | 0.384 | 9.97 | 2,106 | **0.0001** |
| All parasites | 0.263 | 13.64 | 1,106 | **0.0003** |
| All parasites SQRD | 0.189 | 9.83 | 1,106 | **0.0022** |
|  |  |  |  |  |
| **CF** |  |  |  |  |
| Total parasite effects (abundance) |  |  |  |  |
| Season | 0.375 | 4.46 | 2,106 | **0.0138** |
| Lake | 0.645 | 7.66 | 2,106 | **0.0008** |
| All parasites | 0.097 | 2.31 | 1,106 | 0.1314 |
| All parasites SQRD | 0.081 | 1.92 | 1,106 | 0.1683 |
|  |  |  |  |  |
| Total parasite effects (biomass) |  |  |  |  |
| Season | 0.302 | 3.80 | 2,106 | **0.0254** |
| Lake | 0.591 | 7.44 | 2,106 | **0.0009** |
| All parasites | 0.346 | 8.71 | 1,106 | **0.0038** |
| All parasites SQRD | 0.324 | 8.16 | 1,106 | **0.0051** |

SS = sum of squares, df = degrees of freedom

Table C. Results of GLM models for scaled mass index (SMI) and Fulton's condition factor (CF) in common bullies (*G. cotidianus*) that include lake, season and total or individual abundance of the least correlated parasite taxa (from Table 3) as explanatory factors. Unlike Table 4, no quadratic terms were permitted in these models. Bold values indicate significance at P< 0.05.

| Body condition and predictors | SS | F | df | P-value |
| --- | --- | --- | --- | --- |
| **SMI** |  |  |  |  |
| Individual parasite effects |  |  |  |  |
| Season | 0.309 | 7.57 | 2,103 | **0.0008** |
| Lake | 0.363 | 8.88 | 2,103 | **0.0002** |
| *Apatemon* sp. | 0.013 | 0.61 | 1,103 | 0.4348 |
| *Telogaster opisthorchis* | 0.036 | 1.78 | 1,103 | 0.1848 |
| *Tylodelphys* sp. | 0.037 | 1.81 | 1,103 | 0.1810 |
| *Eutrongylides* sp. | 0.057 | 2.81 | 1,103 | 0.0965 |
| *Coitocaecum parvum* | 0.048 | 2.36 | 1,103 | 0.1271 |
|  |  |  |  |  |
| Total parasite effects |  |  |  |  |
| Season | 0.292 | 6.91 | 2,107 | **0.0005** |
| Lake | 0.343 | 8.11 | 2,107 | **0.0015** |
| All parasites | 0.081 | 3.83 | 1,107 | 0.0528 |
|  |  |  |  |  |
| **CF** |  |  |  |  |
| Individual parasite effects |  |  |  |  |
| Season | 0.476 | 5.64 | 2,103 | **0.005** |
| Lake | 0.577 | 6.82 | 2,103 | **0.002** |
| *Apatemon* sp. | 0.056 | 1.32 | 1,103 | 0.253 |
| *Telogaster opisthorchis* | 0.017 | 0.39 | 1,103 | 0.532 |
| *Tylodelphys* sp. | 0.064 | 1.50 | 1,103 | 0.223 |
| *Eutrongylides* sp. | 0.058 | 1.36 | 1,103 | 0.245 |
| *Coitocaecum parvum* | 0.050 | 1.19 | 1,103 | 0.278 |
|  |  |  |  |  |
| Total parasite effects |  |  |  |  |
| Season | 0.466 | 5.36 | 2,107 | **0.003** |
| Lake | 0.524 | 6.16 | 2,107 | **0.006** |
| All parasites | 0.008 | 0.18 | 1,107 | 0.671 |

SS = sum of squares, df = degrees of freedom
